# EpiCRISPR targeted methylation of Arx gene initiates transient switch of mouse pancreatic alpha to insulin-producing cells

**DOI:** 10.3389/fendo.2023.1134478

**Published:** 2023-03-16

**Authors:** Marija Đorđević, Peter Stepper, Clarissa Feuerstein-Akgoz, Clarissa Gerhauser, Verica Paunović, Anja Tolić, Jovana Rajić, Svetlana Dinić, Aleksandra Uskoković, Nevena Grdović, Mirjana Mihailović, Renata Z. Jurkowska, Tomasz P. Jurkowski, Jelena Arambašić Jovanović, Melita Vidaković

**Affiliations:** ^1^ Department of Molecular Biology, Institute for Biological Research “Siniša Stanković” - National Institute of Republic of Serbia, University of Belgrade, Belgrade, Serbia; ^2^ Institute of Biochemistry and Technical Biochemistry, University of Stuttgart, Stuttgart, Germany; ^3^ Division of Epigenomics and Cancer Risk Factors, German Cancer Research Center (DKFZ), Heidelberg, Germany; ^4^ Institute of Microbiology and Immunology, Faculty of Medicine, University of Belgrade, Belgrade, Serbia; ^5^ School of Biosciences, Cardiff University, Cardiff, Wales, United Kingdom

**Keywords:** Arx gene, CRISPR/dCas9, diabetes, pancreatic alpha cells, epigenetic editing, targeted DNA methylation

## Abstract

**Introduction:**

Beta cell dysfunction by loss of beta cell identity, dedifferentiation, and the presence of polyhormonal cells are main characteristics of diabetes. The straightforward strategy for curing diabetes implies reestablishment of pancreatic beta cell function by beta cell replacement therapy. Aristaless-related homeobox (Arx) gene encodes protein which plays an important role in the development of pancreatic alpha cells and is a main target for changing alpha cell identity.

**Results:**

In this study we used CRISPR/dCas9-based epigenetic tools for targeted hypermethylation of Arx gene promoter and its subsequent suppression in mouse pancreatic αTC1-6 cell line. Bisulfite sequencing and methylation profiling revealed that the dCas9-Dnmt3a3L-KRAB single chain fusion constructs (EpiCRISPR) was the most efficient. Epigenetic silencing of *Arx* expression was accompanied by an increase in transcription of the insulin gene (*Ins2*) mRNA on 5^th^ and 7^th^ post-transfection day, quantified by both RT-qPCR and RNA-seq. Insulin production and secretion was determined by immunocytochemistry and ELISA assay, respectively. Eventually, we were able to induce switch of approximately 1% of transiently transfected cells which were able to produce 35% more insulin than Mock transfected alpha cells.

**Conclusion:**

In conclusion, we successfully triggered a direct, transient switch of pancreatic alpha to insulin-producing cells opening a future research on promising therapeutic avenue for diabetes management.

## 1 Introduction

Loss of beta cell identity, dedifferentiation, and the presence of polyhormonal cells stand out as important marker of beta cell dysfunction in diabetic patients. Thus, compensation for the number of pancreatic beta cells can be considered as a therapeutic strategy directly affecting the cause of the disease. Islet transplantation, stimulation of pancreatic beta-cell proliferation, differentiation from embryonic stem cells (ESCs) and cellular reprogramming of other endocrine or exocrine cell types in pancreas (1) could provide a long-term solution in diabetes treatment (2). Unfortunately, the requirement for systemic immune suppression to control immune-mediated rejection of transplanted islets and the limited human islet supply represent significant barriers to progress in this direction (3, 4). By avoiding pluripotent states with their associated malignancy risk, a trans-differentiation approach appears to be safer than approaches based on either ESCs or induced pluripotent stem (iPS) cells (5, 6). On the other hand, promising results were obtained for reprogramming of cells from different origin (7–10) into insulin-producing cells or for initiation of beta cell proliferation (11). These data provide a strong basis for further investigation paving the way for their successful application in the treatment of diabetes.

After extreme beta cell ablation, adult pancreatic alpha cells are able to naturally transdifferentiate and account for a large fraction of newly generated insulin-producing cells (12). The potential of alpha cells to transdifferentiate theoretically resides in a large number of genes bivalent marked by activating H3K4me3 and repressing H3K27me3 histone modifications, while they are in a monovalent state in beta cells (13). Prior studies demonstrated that the transcription factors PAX4, MAFA, NKX6-1, and PDX1, the proinsulin-processing enzyme PCSK1/3 and in mice, the glucose transporter encoded by Slc2a2 are essential regulators of beta cell fate and mature function (14). By contrast, mouse and human islet alpha cells require Aristaless-related homeobox (Arx) to specify cell fate and maintain production of hallmark factors like glucagon (15, 16). Recently published data provided evidence that the expression of Pdx1 and MafA reprogram alpha cells into beta cells *in vivo* in mice and *in vitro* in humans (17). The selective inhibition of Arx in alpha cells or ectopic expression of Pax4 leads to the regeneration of insulin-producing beta cells arising from alpha cells which results in the alleviation of diabetes symptoms in mice whose beta cells have been chemically damaged (18, 19). In addition, deletion of Arx gene (*Arx*) from embryonic stages led to the development of polyhormonal cells (20).

Pancreatic islet epigenetic regulation by DNA methylation appears to be an important regulatory mechanism during alpha and beta cell differentiation and maturation (21–24). Simultaneous inactivation of *Arx* and *Dnmt1* in mouse alpha cells promotes efficient conversion of alpha cells into progenitor cells that established insulin production and secretion, global gene expression and electrophysiology properties in response to glucose stimulation (25, 26). Studies of glucagon^+^ cells in islets from a subset of humans with T1D similarly reveal loss of ARX and DNMT1, with a gain of beta cell features (25).

Many epidrugs altering different epigenetic marks have been developed recently to treat a variety of human diseases, including cancer, diabetes, autoimmunity and genetic disorders (27, 28). However, besides epidrugs that nonspecifically affect any epimark they are designed for, new synthetic epigenetic tools are designed to specifically target certain epigenetic modifications with subsequent effect on the expression of targeted genes (29–33). As a result, epigenome editing has begun to show extraordinary potential in a variety of fields, ranging from basic research to applied biotechnology and has greatly expedited the progress of gene editing from concept to clinical practice (34, 35). A CRISPR/Cas9-engineered INS-1 beta cell line was successfully applied to define the pharmacology of dual GIPR/GLP-1R agonists that target multiple receptors demonstrating the broad utility of CRISPR/Cas9 for the development of potentially novel therapeutics for diabetes treatment (36). Lack of significant difference in glucose tolerance between genetically edited Cre mice and wild-type suggested that the CRISPR/Cas9 methodology provides cell-specific targeting for genetic manipulation of pancreatic beta cells (37). Recently, a robust CRISPR/Cas9 target gene activation (TGA) technology promoted *in vivo* trans-differentiation of liver cells into insulin-producing cells and ameliorated hyperglycemia by increasing serum insulin levels in STZ-treated diabetic mice (38). However, robust CRISPR/Cas9 TGA system induced epigenetic remodeling indirectly by recruiting the transcriptional machinery and by modulating histone marks, while not by directly recruiting epigenetic modulators and editing DNA sequences.

Here we showed that targeted methylation of *Arx* promoter and its subsequent gene silencing unequivocally triggers pancreatic alpha cells to produce insulin. As a tool we used dCas9-Dnmt3a3L-KRAB construct (henceforth EpiCRISPR) and showed that methylation driven-downregulation of just one gene essential for phenotypic expression of alpha cells can stimulate insulin production. This proof of concept has to be further tested in mouse model *in vivo* to be considered as potential therapeutic avenue.

## 2 Results

### 2.1 Transcriptome analysis of mouse pancreatic alpha and beta cell lines

**Figure 1:**
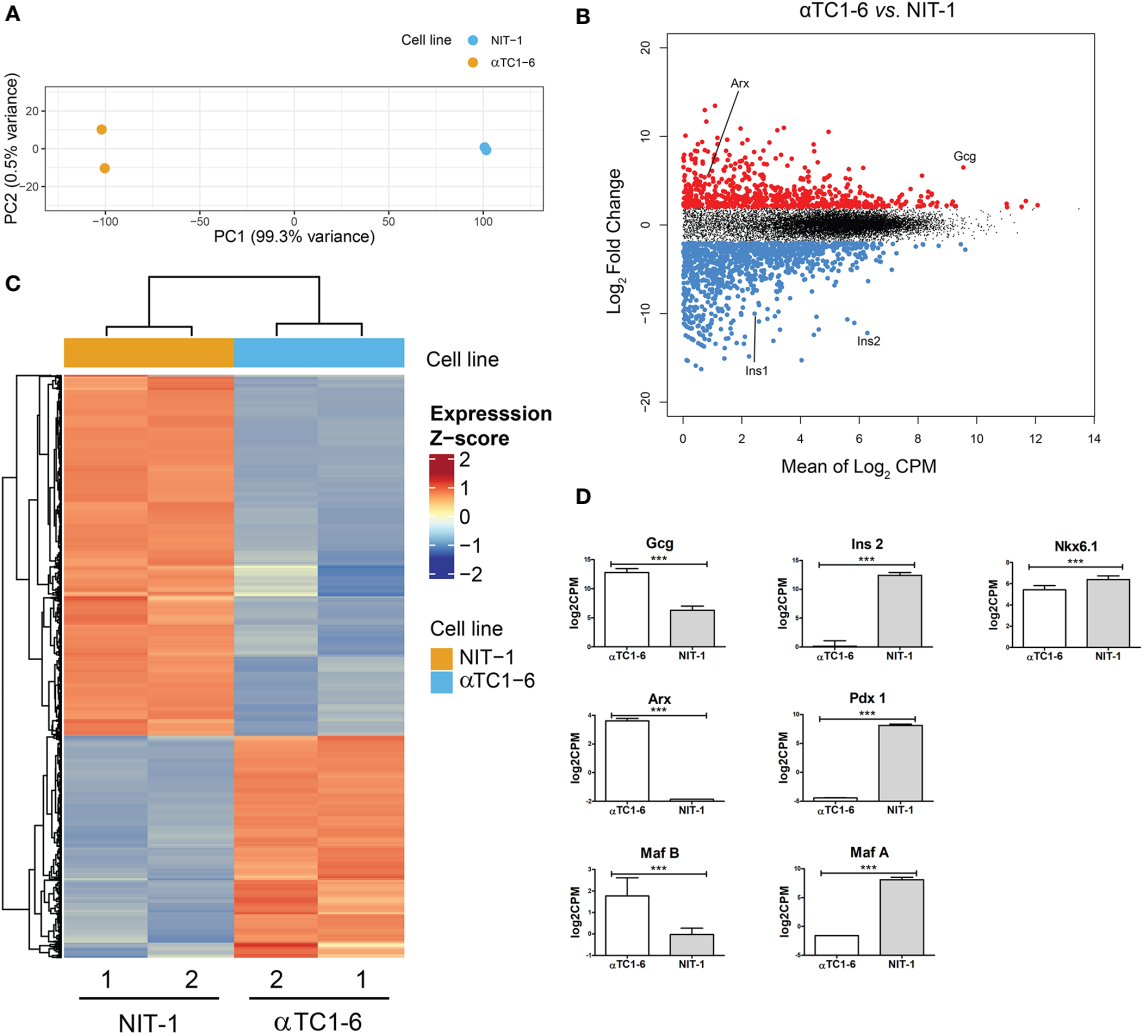
Mouse pancreatic alpha and beta cell transcriptome analysis - characterization of a model system. **(A)** Principal Component Analysis (PCA) of RNA-seq data of an alpha-cell line (αTC1-6, N=2, blue) and a beta-cell line (NIT-1, N=2, orange) describing >99% of the transcriptional variability at the first principal component (N represents the number of biological replicas). **(B)** Log ratio vs. mean average (MA) plot of RNA-seq data displays gene-wise log2 fold change of alpha (N=2) vs. beta-cells (N=2) normalized-averaged counts against mean expression values. Significantly higher (N=654) and lower (N=1061) expressed genes in αTC1-6 vs. NIT-1 cells are highlighted in red and blue, respectively, and display a mean log2 CPM > 0 and a log2 fold change > 0.5 (FDR < 0.05). **(C)** Heatmaps and dendrogram showing the 1715 significantly differentially expressed genes between an alpha-cell line (αTC1-6, N=2) and a beta-cell line (NIT-1, N=2) (see **B**). Heatmap displays gene expression z-score in a color scale between blue and red and samples and genes are clustered by Euclidean distance. **(D)** Expression of selected genes with statistically significant different expression in αTC1-6 vs NIT-1 cells as key factors for maintaining cell identity. p-value: ***p ≤ 0.001.

First, we aimed to identify differentially expressed genes that define the mouse pancreatic alpha and beta cell lines. For this, we completed RNA-seq analysis of murine alpha (αTC1-6) and beta-cell lines (NIT-1) (two biological replicates per each cell line) (**Figure 1**). Principal Component Analysis (PCA) (**Figure 1A**) of alpha and beta cell lines’ transcriptomes using all detected genes (FPKM ≥ 1; N = 2) separated samples into two cell-specific clusters, highlighting that the majority of the variability captured in the transcriptomic data is attributable to the cell-type-specific gene expression patterns (**Figure 1C**). Differential gene expression analysis identified 654 genes with higher and 1061 with lower expression, respectively, in alpha vs. beta cells (**Figure 1B**). Genes specifically expressed in αTC1-6 included classic alpha cell maturation transcription factor (TF) genes such as *Arx* (log_2_ CPM 3.62) and MafB (log2 CPM 1.77). Top differentially expressed (DE) genes in αTC1-6 included the glucagon-encoding gene, *Gcg* without any trace of *Ins1/2* expression. The beta cell line, NIT-1, showed increase expression in genes encoding established beta cell TFs: *Nkx6‑1* (log_2_ CPM 6.39), *Pdx1* (log_2_ CPM 8.10) and *Maf*A (log_2_ CPM 8.09). The top DE genes in NIT-1 included rodent insulin-encoding genes (*Ins1* and *Ins2*) (**Figure 1D**).

**Figure 2:**
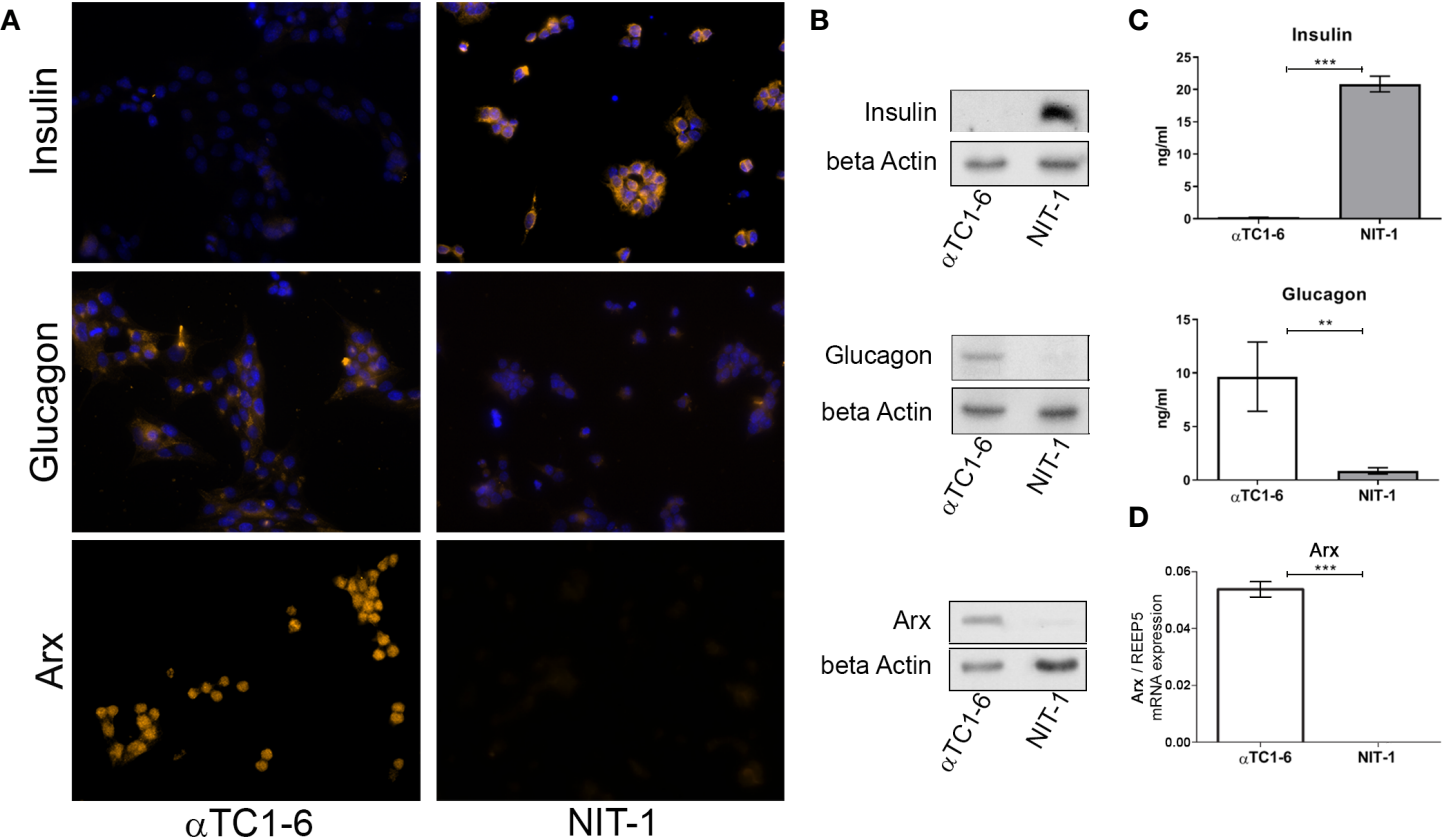
Insulin and glucagon as the main functional cell-type-specific products. **(A)** Immunofluorescence analysis of αTC1-6 and NIT-1 cells with anti-insulin, anti-glucagon, and anti-Arx antibody (light orange fluorescence). Nuclei were stained with DAPI (blue fluorescence). DAPI was not added to cells labeled with anti-Arx antibody. **(B)** Protein expression of insulin, glucagon, and Arx in lysates isolated from αTC1-6 and NIT-1 cells were determined with immunoblot analysis using anti-insulin, anti-glucagon, anti-Arx, and anti-β-actin (loading control) antibodies. **(C)** The amount of secreted insulin and glucagon in cell culture media was measured by enzyme-linked immunosorbent assay (ELISA). **(D)** The relative expression level of *Arx* mRNA isolated from αTC1-6 and NIT-1 cells was determined by RT-qPCR analysis. The *REEP5* mRNA level expression was used as an endogenous control. Data are displayed as mean ± SDs. The error bars denote SD from three biological replicates performed in technical duplicates. Significance among cell type samples was determined using an unpaired Students t-test, **p ≤ 0.01, ***p ≤ 0.001.

Since we intended to use the αTC1-6 cell line as a model system for cellular reprogramming into insulin producing cells, both, mouse beta NIT-1 and alpha αTC1-6 cell lines were further subjected to immunofluorescence (**Figure 2A**) and immunoblot analysis (**Figure 2B**) in order to assess the expression of insulin and glucagon at the protein level. Indeed, as expected the αTC1-6 cells, as terminally differentiated cell type, produce only glucagon, but not insulin. The endocrine functionality of the pancreatic cells was confirmed through their ability to secrete uniquely synthesized and stored hormones. Enzyme-linked immunosorbent assay (ELISA) confirmed that NIT-1 cells release insulin (20.85 ng/ml) in the cell medium without further glucose stimulation in addition to traces of glucagon (0.87 ng/ml). In contrast, αTC1-6 cell line secreted only glucagon (9.67 ng/ml), while no insulin was detected in the cell medium (**Figure 2C**). Next, we examined the presence of Arx protein in both cell lines by immunocytochemistry and immunoblot analysis with anti-Arx antibody and confirmed that Arx is exclusively expressed in αTC1-6 cells but not in NIT-1 cells (**Figures 2A, B**). We also examined *Arx* mRNA expression levels by RT-qPCR. *Arx* transcript levels were confirmed to be statistically significantly higher in αTC1-6 cells compared to NIT-1 cells (**Figure 2D**). This is in agreement with the fact that Arx is a crucial transcription factor necessary to maintain alpha cell identity and is not required for pancreatic beta cells’ maintenance.

### 2.2 The partial epigenetic landscape of *Arx* promoter

**Figure 3:**
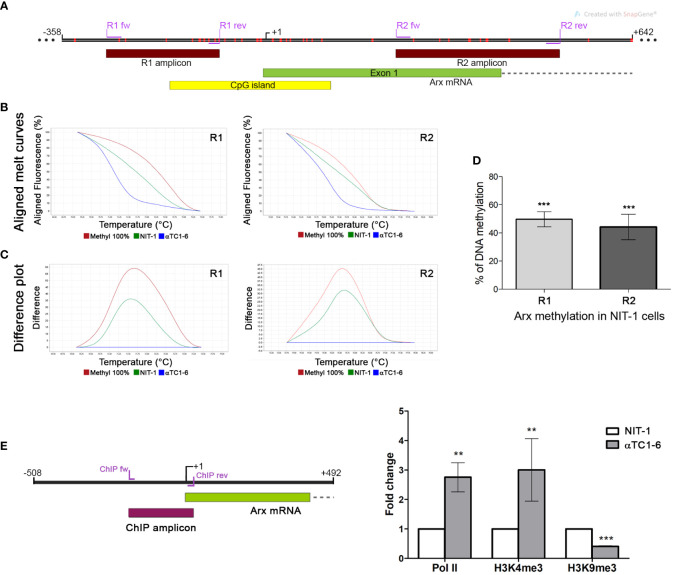
Presence of different epigenetic signatures in the *Arx* promoter of αTC1-6 and NIT-1 cell lines. **(A)** Schematic representation of the Arx genomic region on chr. X with the positions of primers used for DNA methylation analysis shown as purple lines, analyzed parts of the gene shown as dark red boxes, the part of *Arx* mRNA shown as the green box and the position of CpG island shown as the yellow box in the insert. Red vertical lines represent CpG sites in the *Arx* gene. The figure was created with the SnapGene. **(B)** The representative aligned melting curves and **(C)** the difference plots obtained by HRM analysis show positions of NIT-1 curves considering αTC1-6 cells as a 0% standard and commercially methylated mouse DNA standards assumed to be 100% methylated. **(D)** The column chart represents the relative level of DNA methylation in NIT-1 cells in two analyzed regions of the *Arx* gene compared to αTC1-6 cells methylation level (N=3). The results are expressed as means ± SDs. For determining statistical significance the one sample t-test was used, ***p ≤ 0.001. **(E)** ChIP-qPCR analysis of RNA pol II, H3K4me3, and H3K9me3 histone modification occupancy at the *Arx* promoter region (ChIP amplicon) in αTC1-6 and NIT-1 cell lines. ChIP was performed with antibodies against RNA pol II, H3K4me3 and H3K9me3 (N=3). The immunoprecipitated chromatin fragments were analyzed by quantitative PCR using primers for the *Arx* promoter sequence in αTC1-6 and NIT-1 cell lines. The positions of primers used for chromatin ChIP analysis for the *Arx* are represented as purple lines. The results are expressed as means ± SDs. The Kolmogorov-Smirnov test (with D-W-L P value) was used for determining the normality of the ChIP sample. The one sample t-test was used for determining statistical significance values with normal distribution. For data with non-normal distribution, the Wilcoxon Signed Paired test was applied, **p ≤ 0.01, ***p ≤ 0.001.

To confirm that epigenetic mechanisms underlie the observed different expression patterns of Arx as a key regulator of alpha cell identity between the two selected pancreatic cell lines (**Figures 2B, D**), we analyze the DNA and histone methylation pattern of the *Arx* promoter. Firstly, the DNA methylation pattern of two selected regions in the promoter and gene body of *Arx* was analyzed by High-Resolution Melting (HRM). The first analyzed region (R1 amplicon) includes the *Arx* promoter sequence and is located 81 bp upstream of the transcription start site (TSS), providing information about the different DNA methylation status of 10 CpGs in the selected region between αTC1-6 and NIT-1 cells (**Figures 3A, D**). The second analyzed region (R2 amplicon), located downstream (229 bp) of TSS, includes part of the first exon and intron and contains 9 CpGs (**Figures 3A, D**). The TSS track labeled as +1 represents the experimentally validated promoter generated by the Eucaryotic promoter database (EPD) for *M. musculus* (39).

The HRM analysis confirmed significant differences between analyzed cell lines. The column chart denotes a relative level of DNA methylation in NIT-1 cells expressed as a percentage of methylation level between αTC1-6 cells taken as unmethylated [as it was previously shown that Arx promoter is unmethylated in alpha pancreatic cells (23)] and fully methylated DNA standard assumed to be 100% methylated. The open chromatin structure at *Arx* gene promoter and its low methylation profile has been already reported (40, 41) (**Supplementary Figure 1**). Aligned melt curves and difference plots (**Figures 3B, C**) show that the DNA methylation level for both analyzed regions, R1 and R2 in NIT-1 cells is halfway between αTC1-6 cells expressing *Arx* and *in vitro* fully methylated standard. Presented results indicated that the promoter sequence of the *Arx* in NIT-1 cells is more methylated than in αTC1-6 cells for 50% in the first analyzed R1 region and 44% in the R2 region.

Consistent with the RNAseq analysis, control ChIP experiments for RNA pol II antibody confirmed that RNA pol II was more abundantly present on the *Arx* promoter of αTC1-6 than in NIT-1 cells in the analyzed *Arx* promoter region (ChIP amplicon) that encompasses 214 bp including the TSS (**Figure 3E**). The obtained results are referring to the transcriptional activity of the *A*rx gene that corresponds to the *Arx* expression profile in analyzed cell lines (**Figures 2B, D**). In agreement with the transcriptional activity and RNA pol II occupancy, H3K4me3 is significantly more presented in the *Arx* promoter sequence in αTC1-6 cells than in NIT-1 cells (**Figure 3E**) indicating open chromatin structure and transcriptional activity. Conversely, H3K9me3, as a marker of the repressive state of the gene that is intertwined with DNA methylation is more presented in analyzed region in NIT-1 cells than in αTC1-6 cells (**Figure 3E**). These results suggest that the mechanism that regulates *Arx* expression in αTC1-6 and NIT-1 cells includes DNA methylation holding the *Arx* promoter region locked in NIT-1 cells.

### 2.3 EpiCRISPR efficiently introduces targeted methylation of *Arx* in αTC1-6 cells

**Figure 4:**
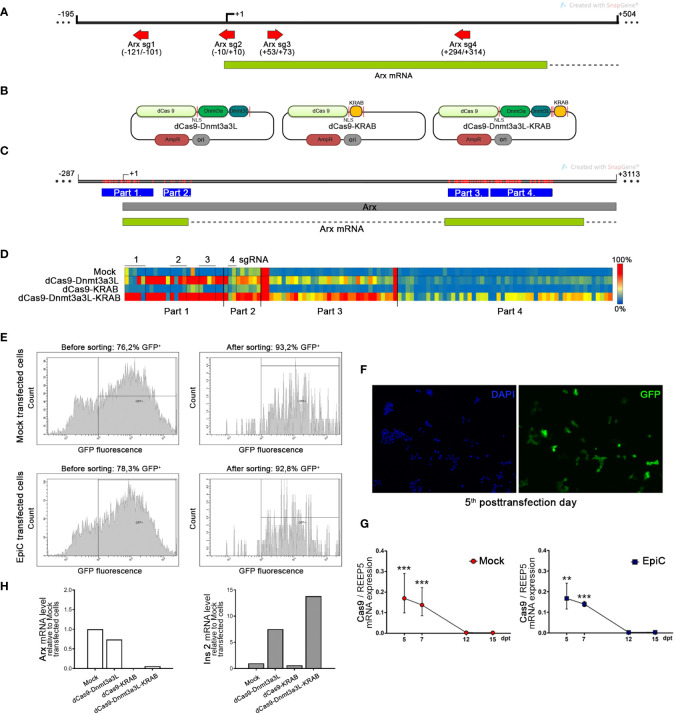
Targeted methylation of the *Arx* promoter in αTC1-6 cells induced by epigenetic editing tool. **(A)** The map shows the position of the four sgRNAs represented as red arrows which were used for transfection and targeting EpiCRISPR fusion construct. The Arx transcribed region is shown as a green box and the intron as a dashed line. **(B)** Schematic representation of used fusion constructs for targeted gene repression. The catalytically inactive dCas9 gene was fused to three different domains with a 28 amino acid linker containing NLS peptide. Not drawn to scale. **(C)** The map shows the part of the X chromosome with the Arx gene indicated as a grey box and with the position of analyzed parts (blue boxes) by bisulfite sequencing in the Arx gene. Red lines represent CpG sites in the Arx gene. **(D)** The targeted DNA bisulfite sequencing analysis represented by the heat map shows the methylation average per CpG site in the Arx promoter sequence. Rows in the heatmap denote separate αTC1-6 co-transfection experiments with one of three different fusion constructs for targeted gene repression in combination with four sgRNAs, while columns represent separate CpG sites in the analyzed region (red-methylated, blue-unmethylated CpG). **(E)** The *fluorescence-activated cell sorting* (FACS) results of transiently transfected αTC1-6 cells before and after cell sorting shows an enrichment of the proportion of fluorescent cells. **(F)** Representative fluorescent microscopy images showed the GFP expression level (green fluorescence) which corresponds to the high transfection efficiency of αTC1-6 cells on the 5^th^ post-transfection day. Nuclei were stained with DAPI (blue fluorescence). **(G)** The relative *Cas9* mRNA expression level in GFP^+^ sorted transfected cell population at 5^th^, 7^th^, 10^th^, 12^th,^ and 15^th^ days after transfection (N=3). GFP – green fluorescent protein; Mock transfected cells – cells transfected with a combination of *pmaxGFP™ Vector*, a plasmid for epigenome editing (dCas9-3a3L-KRAB), and empty gRNA vector; EpiC transfected cells - cells transfected with a combination of *pmaxGFP*™ *Vector*, the plasmid for targeted gene repression and four sgRNAs for targeting Arx gene promoter (Arx sgRNA 1-4 vectors). **(H)** The relative mRNA expression level of *Arx* and *Ins2* on the 5^th^ post-transfection day was determined by RT-qPCR analysis (N=2). The *REEP5* mRNA level expression was used as an endogenous control. The results are expressed as means ± SDs. The Kolmogorov-Smirnov test (with D-W-L P value) was used for determining the normality of the samples. The one sample t-test was used for determining statistical significance values with normal distribution. For data with non-normal distribution, the Wilcoxon Signed Rank test was applied, **p ≤ 0.01, ***p ≤ 0.001.

The αTC1-6 cells were nucleofected with three different fusion constructs for targeted *Arx* repression (dCas9-Dnmt3a3L, dCas9-KRAB, dCas9-Dnmt3a3L-KRAB) (**Figure 4B**), a GFP bearing plasmid and four different sgRNAs (Arx sg1-4). The red arrows in **Figure 4A** show the four used sgRNAs directions and the position relative to TSS. On the 5^th^ post-transfection day, DNA and RNA were isolated from GFP^+^ sorted cells (**Figures 4E, F**). Bisulfite sequencing analysis included the *Arx* promoter region and parts of first and second exons analyzing 117 CpGs in total (**Figure 4C**). Targeted bisulfite sequencing showed that *Arx* promoter was the most efficiently methylated in αTC1-6 cells transfected with dCas9-Dnmt3a3L-KRAB construct (henceforth EpiCRISPR), while no methylation change was observed for cell transfected with dCas9-KRAB construct (**Figure 4D**). The construct dCas9-Dnmt3a3L introduced methylation within Part 1 and 2 of *Arx* promoter region. The best performing construct EpiCRISPR, exhibited methylation rate of 110 out of 117 analysed CpGs.

Transfection with the dCas9 construct fused to the Dnmt3a3L effector domain resulted in the initiation of *Ins2* transcription and insulin synthesis in αTC1-6 cells (**Figure 4H**). Although the dCas9-KRAB construct doesn’t have the ability to directly introduce DNA methylation, KRAB is an efficient transcriptional repressor and thus caused repression of *Arx* expression by chromatin condensation, however no up-regulation of the *Ins2* was observed. The construct dCas9-Dnmt3a3L had minor influence on *Arx* mRNA transcription level, which resulted in a 7.5-fold increase of the *Ins2* mRNA level (**Figure 4H**). The EpiCRISPR (dCas9-Dnmt3a3L-KRAB) construct achieved the highest degree of DNA methylation (110 CpG of 117 CpG analysed) and *Arx* suppression (mRNA level was 16.7-fold lower) in comparison with Mock transfected cells and provided the highest level of *Ins2* expression (*Ins2* mRNA increased 13.8-fold compared to Mock) compared to other dCas9 fusion constructs (**Figure 4H**). Therefore, the EpiCRISPR construct was selected for all further experiments (EpiC transfection). Although the nucleofection efficiency was high (**Figures 4E, F**), on the 5^th^ day after nucleoporation cells were subjected to cell sorting in order to enrich the pool of transfected cells for further analysis (42). After sorting, we achieved 93.2% GFP^+^ for Mock transfected cells and 92.8% GFP^+^ for EpiC transfected cells (cells transfected with dCas9-Dnmt3a3L-KRAB) (**Figure 4E**). As an indicator of the successful transfection, the relative level of mRNA for *Cas9* was examined. *Cas9* mRNA was found in transfected cells at day 5 post-transfection and began to decline afterwards, while on the 12^th^ post-transfection day *Cas9* expression was no longer detectable in EpiC transfected cells (**Figure 4G**).

### 2.4 Targeted methylation and repression of *Arx* triggers initiation of insulin synthesis

**Figure 5:**
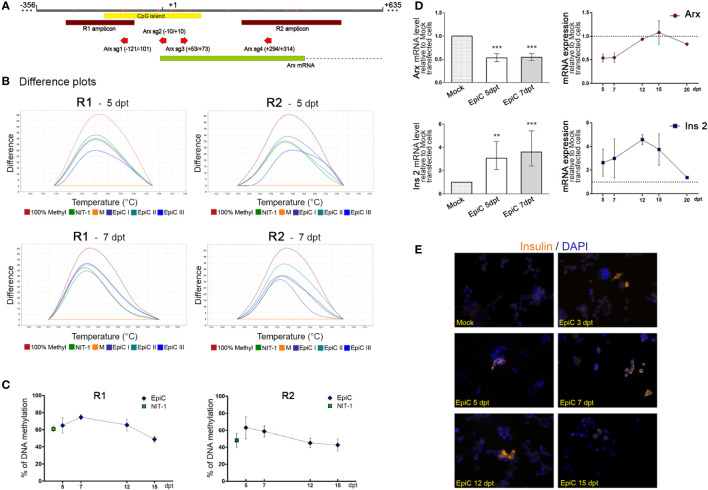
The induced targeted DNA methylation of the *Arx* promotor triggers *Ins2* expression in αTC1-6 cells. **(A)** Schematic representation of a part of the Arx gene with the position of primers used for DNA methylation analysis (R1 and R2 amplicons); the CpG island; and four sgRNAs shown in the inset. Mock-transfected cells with 5% pmaxGFP, 20% dCas9-Dnmt3a3L-KRAB, 75% empty gRNA; EpiC-transfected cells with 5% pmaxGFP, 20% dCas9-Dnmt3a3L-KRAB, 75% all four sgRNAs. **(B)** Representative difference plots obtained from HRM analysis in two different regions of the Arx gene for the 5^th^ and 7^th^ day after transfection shows positions of NIT-1 and EpiC curves in three biological replicates using Mock transfected cells taken as a 0% standard and 100% methylated mouse standard. **(C)** The time scale of changes in DNA methylation level of R1 and R2 analyzed region in EpiC transfected cells relative to Mock transfected cells at 5, 7, 12, and 15^th^ days after transfection. **(D)** The bar chart represents relative *Arx* and *Ins2* mRNA expression levels at the 5^th^ and 7^th^ days post-transfection related to Mock transfected cells. Changes over time in *Arx* and *Ins2* mRNA expression levels at 5, 7, 12, and 15^th^ days after transfection are shown in the line graph (N=4). **(E)** Immunofluorescence analysis of Mock and EpiC transfected cells with anti-insulin antibody (light orange fluorescence) at several days after transfection. Nuclei were stained with DAPI (blue fluorescence). The statistical significance was determined using one sample t-test relative to Mock transfected cells for normally distributed values. The Wilcoxon Signed Rank test was applied for data with non-normal distribution. The error bars denote SD, **p ≤ 0.01, ***p<0.001.

To examine the activity of the transfected plasmid we analyze the DNA methylation pattern in sorted GFP^+^ αTC1-6 cells on the 5^th^ and 7^th^ days after transfection by HRM in the previously (**Figure 3D**) analyzed regions of the *Arx* promoter (**Figure 4**). Mock transfected cells were considered unmethylated and commercially methylated DNA standard was assumed to be 100% methylated. The difference plots (**Figure 5B**) show that the DNA methylation level in the Arx gene of EpiC transfected cells for both analyzed regions (R1 and R2) (**Figure 5A**) at the 5^th^ and 7^th^ day post-transfection reaches a similar level of methylation as it is detected in NIT-1 cells. The line chart indicates that the level of introduced methylation on the *Arx* promoter sequence is comparable to NIT-1 pancreatic beta cells levels. In the first analyzed R1 region at the 5^th^ post-transfection day we detected 65% methylation of EpiC transfected cells and 63% for the R2 region. In both regions methylation was higher than in the Mock transfected cell (**Figure 5C**). On the 7^th^ post-transfection day, we noticed a mild increase in methylation in the R1 region by 9%, while there was a drop in methylation of 4% in R2 region, in comparison to the methylation level on the 5^th^ post-transfection day. In subsequent days, methylation levels continued to decline by ~25%, reaching 40% higher methylation than Mock transfected αTC1-6 cells on 15^th^ post-transfection day (**Figure 5C**). These results confirm that the EpiCRISPR fusion construct efficiently introduced targeted DNA methylation at the promoter region of the *Arx* and is maintained for 7 days after which it starts to decline slowly.

After we efficiently induced targeted DNA methylation in the *Arx* promoter region, we further analyzed the after-effects of changes on *Arx* expression. We detected 50% lower levels of *Arx* mRNA at 5^th^ and 7^th^ day after transfection in EpiC transfected cells in comparison with Mock transfected cells (**Figure 5D**). At the 7^th^ day post-transfection significant reduction in *Arx* mRNA (46%) was observed and a return to the initial level was detected on 12 days after transfection. In parallel with reduced *Arx* mRNA, which occurred as a consequence of EpiCRISPR targeted DNA methylation, EpiC transfected αTC1-6 cells started to produce *Ins2* mRNA on the 5^th^ day post-transfection (**Figure 5D**). The further slightly increased *Ins2* mRNA were detected also on the 7^th^ day post-transfection, the peak was reached on 12 days, followed by a sharp decline in *Ins2* mRNA concentration. On the 20^th^ day after transfection, there was no detected mRNA for *Ins2* in EpiC transfected cells. The immunocytochemistry with anti-insulin antibody confirmed that one part of EpiC transfected αTC1-6 cells became insulin-producing and synthesized insulin have been detected at the protein level from the 3^rd^ to the 15^th^ day after transfection (**Figure 5E**).

**Figure 6:**
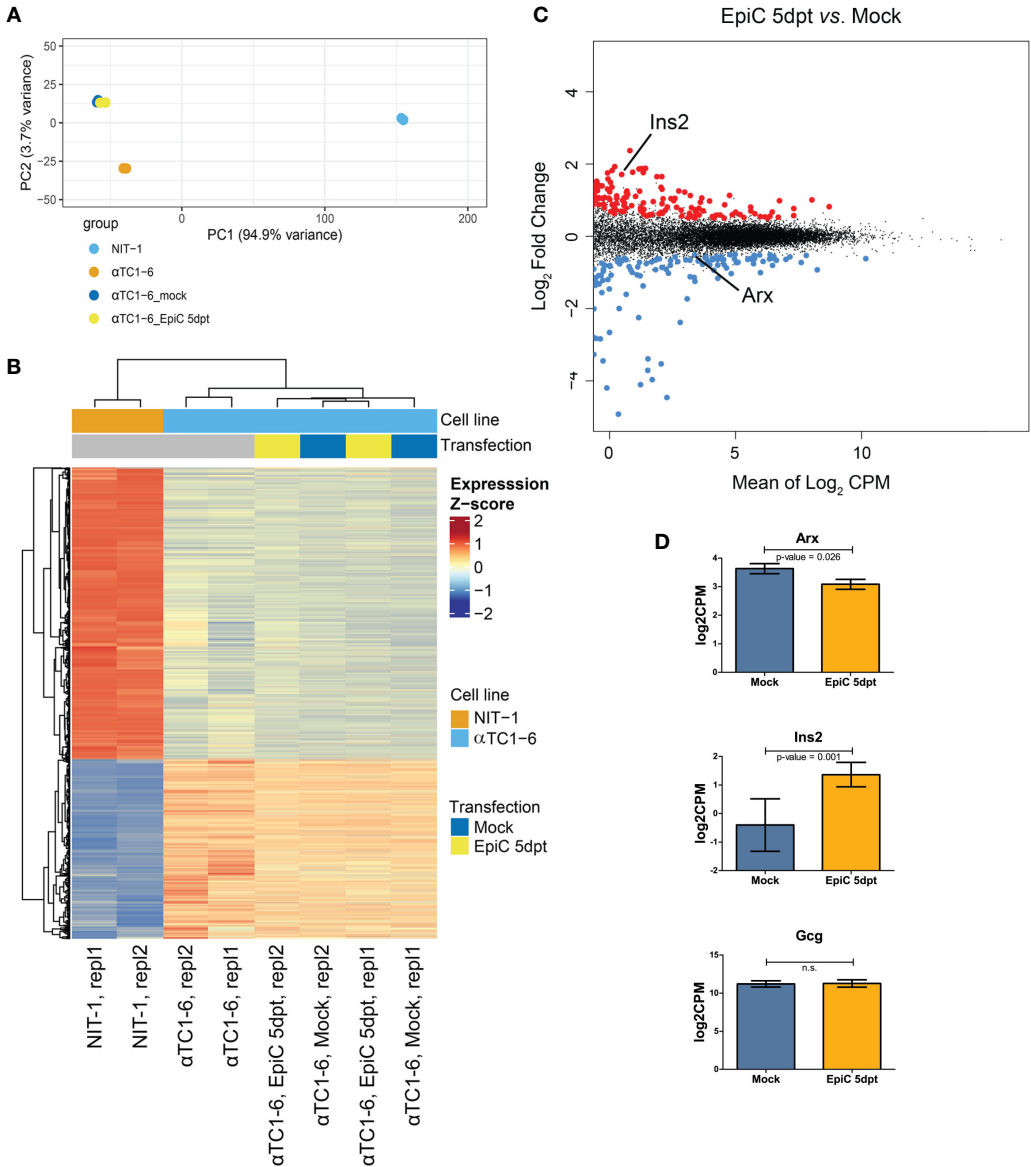
Transcriptomic analysis of Mock and EpiC αTC1-6 cell at 5^th^ post-transfection day. **(A)** PCA of RNA-seq data of αTC1-6 (N=2, orange), NIT-1 (N=2, blue), Mock (N=2, dark blue), and EpiC transfected cells (N=2, yellow), describing >95% of the transcriptional variability at the first principal component. **(B)** Heatmaps and dendrogram showing significantly differentially expressed genes between αTC1-6, NIT-1, Mock and EpiC transfected cells. Heatmap displays gene expression z-score in a color scale between blue and red and samples and genes are clustered by Euclidean distance. **(C)** MA plot of RNA-seq results displays differentially expressed genes of EpiC (N = 2) vs. Mock transfected cells (N = 2). Significantly up- (N = 357) and down-regulated (N = 266) genes are highlighted in red and blue, respectively, and display a log_2_ fold change > 0.5, p-value < 0.05 and mean log_2_ CPM > 0. **(D)** Box plot displays expression differences for *Arx*, *Ins2*, and *Gcg* mRNA expression levels using RNA-seq data. For *Arx* a one-sited and for the other genes two-sided tests were used. ns, not statistically significant.

On the 5^th^ day after transfection, transcriptome analysis by RNA-seq was done on Mock and EpiC αTC1-6 transfected cells (transfected vs. mock, no covariate, logFC > 0.5, pval < 0.05 log2CPM_cutoff > 0) (**Figure 6**). PCA (**Figure 6A**) of alphaTC-1, beta NIT-1, Mock and EpiC transfected cells’ transcriptomes using all detected genes (FPKM ≥ 1; N = 2) separated samples into two cell-specific clusters (alpha and beta-like cells), confirming that EpiC transfected cells still reassemble the gene expression profile of non-transfected alpha cells. Furthermore, control alpha cells are separated from Mock and EpiC transfected cells in a distinct group, highlighting two gene clusters associated with specific up or down regulated genes in all analysed cell lines (**Figures 6A, B**). We detected 357 up- and 266 down-regulated genes in EpiC compared to Mock transfected cells (**Figure 6C**). The significantly elevated mRNA expression of *Ins2* in EpiC transfected cells versus Mock transfection in the transcriptomic analysis corresponds to the elevated *Ins2* mRNA level detected by RT-qPCR analysis. One-sided t-test analysis for *Arx* mRNA expression showed that introduced *Arx* promoter methylation was sufficient to induce the drop in *Arx* expression level (**Figure 6D**).

**Figure 7:**
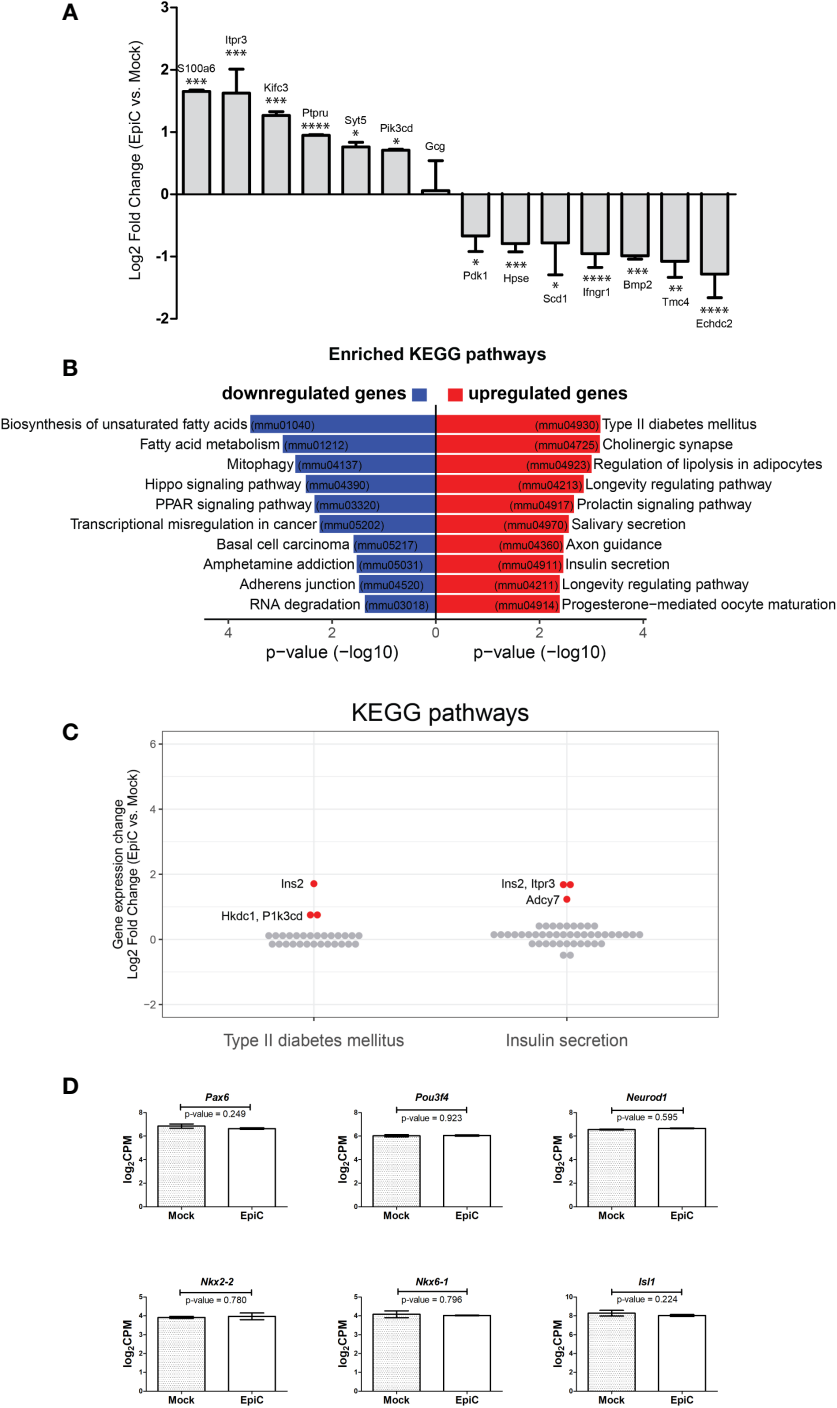
The list of biological processes associated with genes differentially expressed in EpiC vs. Mock transfected cells on the 5^th^ post-transfection day. **(A)** Up- and down-regulated gene in EpiC transfected cells versus Mock displaying log_2_ fold change and p-value: *p ≤ 0.05, **p ≤ 0.01, ***p ≤ 0.001, ****p ≤ 0.0001. **(B)** KEGG pathway analysis. **(C)** Gene expression changes (log_2_ fold change) of all expressed genes involved in Type II diabetes mellitus and Insulin secretion pathways. Significantly differentially expressed genes are highlighted in red (see **Figure 6C**). **(D)** Box plot displays expression differences for *Pax6, Pou3f4, Neurod-1, Nkx2-2, Nkx6-1* and *Isl1* using RNA-seq data. Two-sided tests were used.

The list of up-and down-regulated genes that could be important for shaping beta cell identity indicates a change in expression in genes involved in Ca^2+^ signaling (**Figure 7A**) (for all the genes p-value is less than 0.05), whose expression has to be further analysed at time points later then 5^th^ posttransfection day. We analyzed by KEGG (Kyoto Encyclopedia of Genes and Genomes) pathways analysis which biological processes are associated with genes differentially expressed in EpiC vs. Mock transfected cells at the 5^th^ post-transfection day (pval ≤ 0.05). Genes with upregulation after induced methylation of the *Arx* promoter were associated with a few pathways including the Type II diabetes mellitus and Insulin secretion (**Figure 7B**). Most of the down-regulated genes were associated with the biosynthesis of unsaturated fatty acids and fatty acid metabolism. *Ins2*, *Hkdc1*, and *Pik3cd* were singled out as genes that were expressed to the highest extent in EpiC compared to Mock transfected cells in the Type II Diabetes mellitus pathway while *Itpr3* and *Adcy7* were expressed in the Insulin expression pathway from KEGG analysis (**Figure 7C**) and therefore may represent the next target genes to be examined in the course of alpha cell reprogramming.

Therefore, Arx methylation and lack of the priming factor responsible for pancreatic alpha cell identity is a required minimum for pancreatic alpha cell to start synthesizing insulin. Effect we triggered is not stable and long enough to allow for complete cell reprogramming, but is sufficient for initiation of insulin synthesis. This conclusion is further supported by analysis of pancreatic beta cell specific markers *Pax6, Pou3f4, Neurod-1, Nkx2-2, Nkx6-1* and *Isl1* that are not deferentially expressed between Mock and EpiC transfected cells (**Figure 7D**) confirming that cellular reprogramming is not initiated even dough the phenotypic switch toward insulin production is accomplished.

### 2.5 Epigenetic silencing *Arx* renders αTC1-6 cells become bihormonal

**Figure 8:**
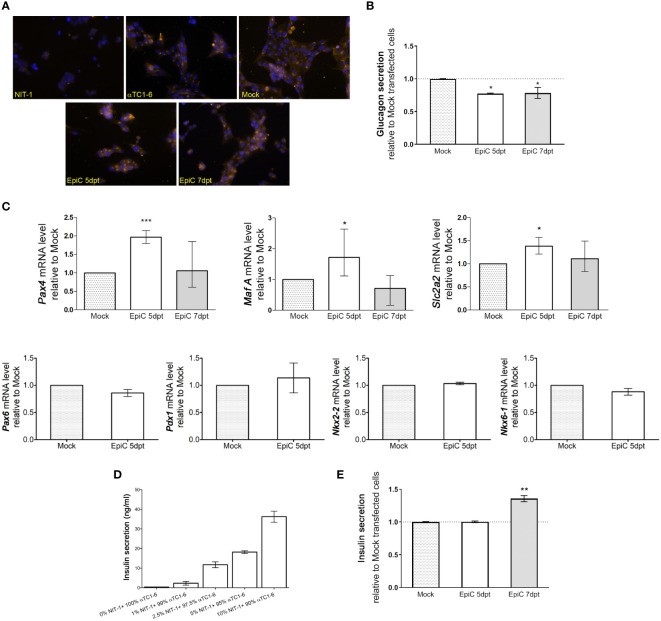
Expression changes in hormone secretion and beta cell-related genes in EpiC transfected αTC1-6 cells. **(A)** Immunofluorescence analysis of Mock and EpiC transfected cells with anti-glucagon antibody (light orange fluorescence) on the 5^th^ and 7^th^ day after transfection. Nuclei were stained with DAPI (blue fluorescence). **(B)** Glucagon secretion in cell culture media was measured by ELISA on the 5^th^ and 7^th^ day after transfection (N=4). **(C)** Relative mRNA expression level of *Pax4*, *MafA* and *Slc2a2* on the 5^th^ and 7^th^ day as well as *Pdx1*, *Pax6*, *Nkx2-2*, *Nkx6-1* on the 5^th^ day after transfection related to Mock transfected cells (N=4). The REEP5 mRNA level expression was used as an endogenous control. **(D)** Glucose-stimulated insulin secretion measured by ELISA assay. The concentration of secreted insulin in cell culture media in αTC1-6 mixed with NIT-1 cell lines in different percentages (0-10%) was determined after adding 30 mM glucose in the KRB buffer. **(E)** Insulin secretion in αTC1-6 cell culture media was measured by ELISA assay on the 5^th^ and 7th day after transfection (N=3). The one sample t-test was used for determining statistical significance, *p ≤ 0.05, **p ≤ 0.01, ***p ≤ 0.001.

An immunocytochemistry experiment with anti-glucagon antibody showed that besides induction of insulin synthesis, there were no obvious changes in glucagon presence in the cells after Mock and EpiC transfection compared to untransfected cells (**Figure 8A**). The quantification of glucagon by ELISA assay showed that the bihormonal EpiC transfected αTC1-6 cells release ~20% less glucagon than Mock transfected cells in cell culture medium on 5^th^ post-transfection day (**Figure 8B**). The analysis of EpiC transfected cells at 5^th^ and 7^th^ day post-transfection showed that there were changes in the transcriptional pattern of the two crucial transcriptional regulators for maintaining beta cells phenotype. Using RT-qPCR we could confirm that mRNAs for two TF, *Pax4*, *MafA*, and for glucose transporter gene, *Slc2a2* were significantly up-regulated in EpiC compared to Mock transfected cells at the 5^th^ post-transfection day, followed by a drop in expression and return to baseline at the 7^th^ post-transfection day (**Figure 8C**). RT-qPCR for *Pax6*, *Nkx6-1*, *Nkx2-2* and *Pdx1* did not show any difference between Mock and EpiC transfected cells (**Figure 8C**). In the line with RNA-seq data, these results confirmed that single transfection approach with EpiCRISPR is able to induce short-term effect on *Arx* suppression and induction of *Ins2* synthesis as a prerequisite for further pancreatic cells reprogramming.

Pancreatic beta cells are essential for energy homeostasis, acting through glucose-sensing mechanisms. Glucose‐stimulated insulin secretion (GSIS) involves a complex regulatory system of pancreatic beta cells for the recognition of extracellular glucose concentration and proper insulin secretion as required at a given time. The validation of the functionality of NIT-1 cells was done by examination of their ability to respond to 30 mM glucose with the extracellular release of insulin. GSIS assay was done in a population of control αTC1-6 cells mixed with different percentages of NIT-1 cells (0%, 1%, 2.5%, 5%, and 10%) for internal standardization of the percentage of insulin-producing cells required for detection of secreted insulin by ELISA assay. The concentration of secreted insulin in KRB buffer was determined 2 h after adding 30 mM glucose by ELISA assay (**Figure 8D**). About 36.2 ng/ml of secreted insulin was measured in control αTC1-6 cells mixed with only 10% of NIT-1 after glucose stimulation. Further dilution was done in order to estimate the lowest measurable level of insulin that could be detected by ELISA. Only 1% of insulin-producing NIT-1 cells in a bulk of 99% of control αTC1-6 could release a measurable level of insulin (2.3 ng/ml) after glucose stimulation. By confirming the minimal amount of insulin to be detected in the cell medium by ELISA, the EpiC transfected cells’ ability to secrete insulin into the growth medium was verified using the same assay (**Figure 8E**). There was no measurable level of insulin secreted in the cell medium on the 5^th^ day after EpiC transfected cells compared to Mock transfection, while it was observed that 7 days after transfection 35% of insulin (3.3 ng/ml) is released from the EpiC cells into the medium if compared to Mock transfected cells. If the amount of secreted insulin in EpiC transfected cells is compared to the same value measured after mixing pancreatic alpha and beta cells (**Figure 8D**), we can conclude that around 1% of EpiC transfected cells were able to secrete insulin and therefore to be defined as insulin-secreting cells.

## 3 Discussion

Bearing in mind that pancreatic alpha and beta cells identity depends on epigenetically controlled antagonistic activities of Arx and Pax4 respectively (15), targeting Arx suppression in pancreatic alpha cells by DNA methylation offers a solution for production of insulin with a minimal intervention and minimal side effects, as in majority of both exocrine and endocrine pancreatic cells Arx is already methylated. A study of Dhawan et al. that triggered our investigations, pointed to pancreatic beta to alpha cell dedifferentiation in response to ablation of the DNA methyltransferase 1 (Dnmt1) in beta cells (23, 24). They identified the lineage determination gene Arx as methylated and repressed in beta cells, and hypomethylated and expressed in alpha cells and Dnmt1-deficient beta cells. In the same year, Papizan et al. demonstrate that Nkx2.2 is part of a large repression complex in pancreatic beta cells indicating that repressor activities of Nkx2.2 on the methylated Arx promoter in beta cells are required for maintaining beta cell identity (21). Our study represents a step forward, since we were exploring the possibility of using an epigenetic editing tool (EpiCRISPR) for “sniper-shot” suppression of the Arx gene by methylation. Using epigenetic editing, we were able to induce insulin synthesis in approximately 1% of transfected alpha cells. This is in agreement with the data of spontaneously reprogrammed 1–2% of alpha cells after massive loss of pancreatic beta cells as a direct consequence of insulin signaling deprivation (43, 44). So far, around 98% of alpha cells refuse to convert and do not spontaneously transit towards a beta-like cell phenotype (45). In our *in vitro* epigenetic editing study, we reached the same percentage of alpha cell switching into insulin producing cells as shown for simultaneous transition of alpha cells under diabetic conditions. Furthermore, the rationale behind the strategy to use pancreatic alpha cells from the same organ (pancreas) for trans-differentiation is the fact that alpha-to-beta cell trans-differentiation can lead to restoration of beta cell mass, but it also simultaneously reduces alpha cell mass and thus restores the balance between pancreatic hormones (insulin and glucagon), which is perturbed in diabetes (bihormonal hypothesis of diabetes) (46). There is a lot of data regarding the restoration of the beta cell mass but little is known about the regulation of alpha cell mass. Recently, Kodani et al. (47) showed that Foxo1 binding to the *Arx* promoter can led to Dnmt3a dissociation and *Arx* promoter hypomethylation, suggesting that the FCoR-Foxo1 axis regulates pancreatic alpha cell mass by suppressing *Arx* expression. The intra-islet plasticity (48) has been suggested to be the mechanism for regulating beta cell and other endocrine cell masses. Therefore, increasing the number of insulin-producing beta cells while decreasing the number of glucagon-producing alpha cells turns out to be a promising therapeutic avenue in diabetes treatment. Impairment of glucagon signaling that leads to a marked increase in alpha cell mass raises the possibility that such alpha cell hyperplasia provides an in-creased supply of alpha cells for their trans-differentiation into new beta cells (49).

All published results strongly indicate that the pattern of genes expressed in different pancreatic islet cells and their epigenetic states need to be maintained after cell division to ensure proper pancreatic islet cell identity. The implication of these findings is that a specific chromatin structure accompanies heritable gene repression (50). This is in complete agreement with our RNAseq analysis of αTC1-6 and NIT-1 cell lines that showed two cell-specific clusters based on the presence of cell-type-specific gene expression patterns. Also, we showed that the epigenetic landscape of the *Arx* promoter corresponds to fully functional gene expression in αTC1-6 cells in contrast to the *Arx* promoter repression in NIT-1 cells. The same results are revealed by Lawrol et al. (40) showing that open chromatin landscapes in the human primary cells and mouse alpha cell line are conserved at *Arx* locus in contrast to beta cell counterpart. Upon visualizing gene expression and chromatin accessibility, Mawla and co-workers (41) also confirmed matching gene expression and TSS chromatin accessibility of key transcription factors known to regulate identity of each analysed pancreatic cell type. *Arx* mRNA expression analyses showed that introducing *Arx* promoter methylation in EpiC transfected alpha cells was sufficient to induce a reduction in *Arx* mRNA expression levels. This led to up- and down-regulation of several genes that could be important for transient switch towards insulin production (*Ins2* and some genes involved in Ca^2+^ signaling). Also, genes that were up-regulated after induced methylation of the *Arx* promoter were associated with a few pathways including Type II diabetes mellitus and Insulin secretion pathways.

To be perfectly clear, we did not initiate cellular reprograming since specific beta cell markers Pdx1 or Neurod1 that has been successfully used to reprogram various cell types into insulin-producing cells *in vitro* and *in vivo* (51) are not found to be differentially expressed in EpiC transfected cells. We only found transient increase in *Pax4*, *MafA* and *Slc2a2* in EpiC transfected cells that started to produce insulin (5^th^ post-transfection day) which decline after one week of culturing. This might be starting point for progressing towards cell reprogramming but in this stage we can claim that suppression of *Arx* by methylation is sufficient for induction of insulin synthesis but not for complete reprogramming towards beta-like cells. In order to initiate full pancreatic alpha cell reprogramming in our further research, we have to provide constant expression of beta cell related TF. The study of Collombat et al. (15) was first to show pancreatic alpha to beta cell trans-differentiation in response to enhanced expression of homeobox protein Pax4. The same interendocrine spontaneous adult alpha to beta cell conversion was observed after extreme ablation of beta cells (12). Furthermore, the ectopic expression of *Pax4* in alpha cells led to restoring a functional beta cell mass and to diabetes cure in animals that were chemically depleted of beta cells (18). Cigliola et al. (43) showed that genetic inhibition of the Smoothened G protein-coupled receptor, together with beta cell loss, enhanced alpha cell reprogramming as well as direct modulation of insulin signaling. Finally, Furuyama et al. (52) revealed that ectopic expression of the TFs PDX1 and MAFA in human alpha cells efficiently converts them into insulin-secreting cells that lead to diabetes reversal when transplanted into diabetic mice.

The CRISPR/Cas9 technology offers straightforward advantages in targeting new sequences and has been harnessed for genome editing in a broad range of organisms and for targeting different diseases (35, 53). All recent publications that reported use of different CRISPR/Cas9 systems for diabetes attenuation were targeting a variety of diabetes-related genes and different cell types: Liao and coworkers (36) succeeded to overexpress Pdx1 in liver cells by CRISPR/Cas9-mediated TGA system which initiated liver cells trans-differentiation into insulin-secreting cells in a mouse model of diabetes. Ou et al. (54) used the TALE-TET1 system for demethylation of the imprinted control region 2 (ICR2), which resulted in increased replication of pancreatic beta cells. Gimenez and coworkers (55) used the CRISPR/dCas9-VP160, CRISPR/dCas9-TET1 and CRISPR/dCas9-P300 systems for multiplex epigenetic editing and activation of human pancreatic beta cell genes (PDX1, NEUROG3, PAX4 and INS) essential for maintaining beta cell identity. We are proposing targeted DNA methylation of the *Arx* promoter as an efficient, precise and reversible approach for gene suppression and initiation of insulin production. We started with 3 different constructs: dCas9-Dnmt3a3L, dCas9-KRAB, dCas9-Dnmt3a3L-KRAB and four different sgRNAs. The EpiCRISPR programmable epigenetic editor system (dCas9-Dnmt3a3L-KRAB) showed the best *in vitro* targeting DNA methylation abilities. After single transient transfection (42), EpiCRISPR-induced hypermethylation and condensation of the promoter region of *Arx* initiated transition of pancreatic alpha to insulin-producing cells. This programmable epigenetic editor system (without KRAB repressor) has been already used in the study of Stepper et al. (32) showing that peaks of targeted methylation were observed around 25 bp upstream and 40 bp downstream of the PAM site, while 20–30 bp of the binding site itself are protected against methylation. The authors proposed that the potent DNA methylation is dependent on the multimerization of Dnmt3a/Dnmt3L complexes on the DNA. Furthermore, the introduced methylation causes transcriptional repression of the targeted genes. Using the same epigenetic editor system Saunderson et al. (56) revealed that DNA methylation can be targeted to multiple genes in primary breast cells isolated from healthy human tissue, resulting in long term hypermethylation and gene silencing. Our EpiCRISPR system besides Dnmt3a3L has KRAB repressor fused to N-terminus of the dCas9, helping in chromatin condensation into the densely packed higher-order structures at the targeted sequence (*Arx* promoter) and thereby additionally represses the gene expression. Similarly to our study, O’Green and coworkers (57) examined the ability of two combined epigenetic toolboxes, DNMT3A-dCas9 and KRAB-dCas9 simultaneous transfected, to induce long-term repression at some target genes at which H3K9me3 and DNA methylation were transiently acquired and subsequently lost. Our single transfection approach enabled one week hypermethylation effect on the *Arx* gene which after 12 days returned to control values. At the same time we detected a peak in *Ins2* mRNA expression at 7^th^ post-transfection day which continue to increase until day 12 post-transfection. After this time point insulin mRNA sharply decline and return to control value for alpha cells. We decided to analyze several time points: 5, 7, 12, 15 and 20 post-transfection days. These time points are matching with the previous observation of Thorel et al. (12) stating that 5 dpDT is a point of alpha cell’s early response to injury, 15 dpDT is the time when the first converted alpha cells are observed and still bihormonal and 30 dpDT is time when converted cells no longer expressing glucagon are present in the islets (3). In order to proceed further towards pancreatic alpha cell reprogramming into beta-like cells we need to improve stability of *Arx* suppression by methylation and to provide more time for cells to undergo reprogramming process by expressing beta cell related genes that could activate different signaling pathways.

This study confirmed that transient transfection of pancreatic αTC1-6 cells with an EpiCRISPR construct, exhibited *Arx* promoter hypermethylation and *Arx* suppression five days post-transfection. At the same time, EpiCRISPR edited cells were confirmed as insulin-producing cells by, immunofluorescent staining of intracellular insulin level and measuring insulin secretion by ELISA assay. Using the EpiCRISPR construct we induced insulin synthesis to a level 35% higher compared to Mock transfected cells. In contrast to the applied single transfection, our future experiments will involve a multiple transfection approach that will enable improved stability of epigenetic marks, resulting in a more pronounced effects on *Arx* suppression (by analogy to “extended gene expression” technique used in prolonged production of recombinant proteins (58)) as well as multiplexing for targeted suppression of *Arx* and activation of *Pax4* (or other beta cell related TF) in alpha cells that will eventually lead to enhanced insulin production and complete cellular reprogramming. This increase in insulin synthesis and secretion is designed to influence the hyperglycemic status and has to be confirmed *in vivo* in diabetic animals.

## 4 Materials and methods

### 4.1 Cell cultures

Mouse pancreatic alpha TC1 clone 6 (αTC1-6, American Type Culture Collection, Manassas, VA, USA, CRL-2934) cell lines were cultured in 15 mM glucose Dulbecco’s Modified Eagle’s medium (DMEM) supplemented with penicillin/streptomycin (Gibco, Invitrogen Corporation, US), 10% fetal bovine serum (FBS) (Sigma) and 0.02% bovine serum albumin (SERVA Electrophoresis GmbH, Heidelberg). Mouse pancreatic beta-cell line NIT-1 was cultured in the Ham’s F-12K Nut Mix (1x) medium with 10% FBS and penicillin/streptomycin. The cell medium was changed every second day. After reaching 70% confluence, cells were propagated by detaching with phosphate-buffered saline (PBS) for cell dissociation without enzyme (GIBCO, by Life Technologies). All cells were grown at 37°C in humidified air containing 5% CO_2_.

### 4.2 Nucleofection and cell sorting

After reaching a confluence of 70%, the αTC1-6 cells were nucleofected using Amaxa™ 4D-Nucleofector™ X Unit. Cells were washed in PBS and detached by cell dissociation buffer. After centrifugation step at 90 x g for 10 min, cells were resuspended in 4D-Nucleofector™ SF Solution with Supplement for nucleofection. The cells were mixed with plasmids, transferred to Nucleocuvette™, and exposed to an electrical pulse. The optimized protocol specifies the CM-156 as a program of choice for αTC1-6 cell line nucleofection in combination with SF Solution (42). Cells were incubated for 10 min at 37°C in RPMI medium as a recovery step after nucleoporation, seeded in 6-well sterile culture plates, and propagated until sorting. All steps for nucleofection included gently handling the cells. The fluorescence-activated cell sorting was used for collection of GFP positive αTC1-6 cells on the 5^th^ and 7^th^ post-nucleofection day by FACS Aria III flow cytometer and cell sorter (BD Biosciences, San Diego, USA). The cells were analyzed by FACS Diva software. Hanks’ buffered saline solution (HBSS) buffer without calcium and magnesium ions supplemented with 2 mM EDTA and 2% FBS was used for the sample preparation for the cell sorting.

### 4.3 Plasmids (constructs)

Set of fusion plasmids for targeted gene repression containing epigenetic effector domains fused to dCas9 (dCas9-Dnmt3a3L, dCas9-KRAB, dCas9-Dnmt3a3L-KRAB) were used for transfection experiments (32, 59). The cells were transfected with fusion plasmid for targeted epigenome editing (20% of total DNA) and 5% of reporter plasmid pmaxGFP™ (supplied in Nucleofector™ Kit) used in combination either with empty gRNA plasmid (Addgene plasmid #41824, gRNA_Cloning Vector, a gift from George Church) (75% of total DNA) for Mock nucleofection or with four different sgRNAs (75% of total DNA) (**Table 1** - **Supplementary Material**) for EpiC nucleofected cells.

### 4.4 Isolation of DNA and RNA

ZR-Duet™ DNA/RNA MiniPrep Kit (Zymo Research, Irvine, CA 92614, USA) was used for the isolation of genomic DNA and total RNA from the cells according to the manufacturer’s instructions. Total RNA from untransfected control cells was isolated using the GeneJET RNA Purification Kit (Thermo Fisher Scientific, USA) according to the manufacturer’s guidelines.

### 4.5 Real‐time quantitative PCR

Total RNA extracted from untransfected and nucleofected cells was subjected to cDNA synthesis with RevertAid First Strand cDNA Synthesis Kit (Thermo Fisher Scientific, USA) using mixed oligo(dT) and random hexamer primers (1:1). The Maxima SYBR Green/ROX qPCR Master Mix (Thermo Fisher Scientific, USA) and The Quant Studio 3 Real‐Time PCR system (Applied Biosystems, Carlsbad, CA, USA) were used for quantification of analyzed mRNA. The thermal cycles involved an initial denaturation step at 95°C for 10 min and 40 cycles of two-step PCR at 95°C for 15 s and 60°C for 60 s. The relative expression level of target genes was calculated by the comparative 2^-ΔΔCt^ method after normalization by REEP5 as endogenous control. The primers were designed in Primer‐BLAST (https://www.ncbi.nlm.nih.gov/tools/primer‐blast/) for murine sequences stored in GenBank. The primers used for analysis are listed in **Table 2** (**Supplementary Material**).

### 4.6 RNA-seq (Transcriptome analysis)

Stranded mRNA-seq libraries were prepared at the Genecore facility (EMBL, Heidelberg, Germany) using Illumina TruSeq RNA Sample Preparation v2 Kit. The libraries were pooled in equimolar amounts and sequenced in a single end setting on the Illumina NextSeq 500 High output machine with 75 bases long reads. Data were processed with the nf-core rnaseq pipeline (60). Default parameters were used unless mentioned otherwise. Sequences were aligned to the mouse reference genome (mm39/GRCm39, Ensemble release 105) by application of the software RSEM (–aligner star_rsem). Normalized counts per million (CPM) were used for statistical analyses performed by edgeR (61). To identify differentially expressed genes, exact test for differences between two groups was applied and gene with a log2CPM > 0, log2 fold change > 0.5 and FDR < 0.05 (alpha vs. beta cell line) or pval < 0.05 (EpiC vs. Mock transfected) was considered as significant. For the target gene (Arx) a single-sided exact test was applied. Principal component analysis was performed on log2CPM values of the 500 most variable genes. Heatmap was generated by ComplexHeatmap on scaled expression levels (z-score) of significantly differentially expressed genes (62). Genes and samples were clustered by complete linkage of the Euclidean distance. KEGG pathway overrepresentation analysis was performed using WebGestalt (WEB-based GEne SeT AnaLysis Toolkit) application (http://www.webgestalt.org) on all significantly up- or downregulated genes using default settings and top ten significantly enriched pathways were displayed (63).

### 4.7 Bisulfite conversion of DNA and primer design

Genomic DNA from control αTC1-6 and NIT-1 cells and nucleofected αTC1-6 cells was bisulfite-converted using the EZ-DNA Methylation™ Kit (D5001; Zymo Research, Irvine, USA) following the manufacturer’s recommendations. The MethPrimer (http://www.urogene.org/cgi-bin/methprimer/methprimer.cgi) and genomic sequence with NCBI ref. sec. NC_000086.7 (Arx gene, assembly: GRCm38.p6 (GCF_000001635.26)) was used for designing primers for high-resolution melting (HRM) analysis. The first set of primers (R1) encompassed sequences from -279 to -81 with regard to the position of TSS marked as +1 and is composed of two pairs of primers targeted to the same location making the difference between the methylation states. One pair of primers was complementary to the methylated and the other to the unmethylated bisulfite-converted target DNA sequence which allows all combinations of methylation status to be covered. The second set of primers (R2) was designed to be complementary to the DNA sequence that does not contain CpG dinucleotide and could not be differentially methylated, covered the sequence from +229 to +516 downstream from TSS.

### 4.8 Methylation-sensitive high-resolution melting

Mouse methylated standard (D5012, Zymo Research) and isolated gDNA from the cells were subjected to bisulfite conversion according to manufacturer’s instructions. Genomic DNA from untreated αTC1-6 was used as unmethylated standard as it was previously shown that *Arx* promoter is unmethylated in alpha pancreatic cells (23). The QuantStudio 3 Real‐Time PCR system (Applied Biosystems) was used for PCR amplification. PCR was performed in 10 µl reaction mixture composed of 5 µl 2x MeltDoctor HRM Master Mix (Applied Biosystems), 0.4 µM of each primer (0.8 µM for R2 set of primer; **Table 3** - **Supplementary Material**) and 2 µl bisulfite converted DNA template (theoretical concentration of 20 ng/µL). The temperature profile for amplification consisted of an initial denaturation at 95°C for 10 min, followed by 40 cycles of three-step PCR and a final elongation step at 72°C for 7 min. Three-step PCR included denaturation at 95°C for 15 s, annealing at 57°C for R1 or 59°C for R2 for 30 s and elongation at 72°C for 30 s. The additional melt curve stage comprised of temperature ramping from 60‐95°C by 0.025°C/s with fluorescence acquisition at each temperature increment. HRM Software v3.1 (Applied Biosystems) was used for end-product analysis. Peak heights, obtained from difference curve aligned against the unmethylated control (0%), were used for calculating the degree of methylation of analyzed samples relative to methylated control (100%).

### 4.9 NGS library preparation and high-throughput sequencing

For bisulfite sequencing, isolated DNA was converted with Zymo EZ DNA Methylation-Lightning Kits according to manufacturer’s recommendations. Regions of interest on the Arx promoter where amplified using Qiagen’s HotStarTaq Polymerase with 2.25 mM MgCl_2_ and 0.033 U/μl polymerase (**Table 4** - **Supplementary Material**) with the primers in final concentration of 300 nM (**Table 5** - **Supplementary material**). The PCR products were run on agarose gels for quality control and cleaned-up using either the NucleoSpin Gel and PCR Clean-up kit (Macherey-Nagel). The SureSelect library preparation kit (Agilent Technologies) were used for end-repair and A-tailing of combined bisulfite amplicons for each sample. The samples were ligated to unique TruSeq HT double indexed adapters, then pooled and amplified using PCR with Q5 polymerase for eight cycles. The NEBNext Library quantification kit for Illumina (NEB) was used for the library quantification, and the clean-ups during the library preparation were achieved by magnetic SPRI beads. The MiSeq machines with 2x300 PE runs were used for libraries sequencing. The optained sequencing results were demultiplexed (Qiime) (64), than quality filtered and adapter trimmed (Trim Galore v0.4.1, using the default parameters with a cut-off at Phred 20 and –paired. The sequences were mapped to the GRCm39 assembly of the mouse genome (Bismark v0.14.4, using –non_directional). The percentage of methylation reads for each CpG site was determined with SeqMonk using the “Difference quantification” and the “Annotated probe report” functions. The final analysis was done using Microsoft Excel.

### 4.10 Immunoblot analysis

αTC1-6 and NIT-1 cells were lysed in ProteoJET™, a mammalian cell lysis Reagent (Fermentas, Life science) supplemented with a protease inhibitor cocktail for 30 min at 4°C. Equal amounts of cell lysates were separated by 9% (for Arx analysis)-15% (for Insulin and Glucagon analysis) sodium dodecyl sulfate polyacrylamide gel electrophoresis and transferred onto polyvinylidene difluoride membranes (Amersham Hybond P 0.45 PVDF, GE Healthcare Life Sciences). Immunoblotting was performed overnight by incubation at 4°C with the primary antibody, followed by incubation with the appropriate horseradish peroxidase-conjugated secondary antibody at room temperature for 60 min. All used antibodies and their dilutions are listed in **Table 6** (**Supplementary Material**). Detection was performed by the enhanced chemiluminescence detection system according to the manufacturer’s instructions (Amersham Pharmacia Biotech, Amersham, UK). The intensities of the signals were quantified using TotalLab electrophoresis software, ver. 1.10 (Phoretix, Newcastle upon Tyne, UK).

### 4.11 Immunocytochemistry

Cells were seeded on sterile glass coverslips in 24-well tissue culture plates. Control cells were fixed after reaching 70% of confluency, while nucleofected cells were fixed at a few different time points after nucleofection. Cells were fixed with 4% paraformaldehyde (Science Services GmbH, Munich, Germany) in PBS for 10 min at room temperature (RT) and permeabilized in the 0.3% Triton X-100 in PBS for 10 min at RT. The blocking was done in 3% bovine serum albumin in PBS or 10% normal donkey serum for 60 min at RT. A list of the used primary and secondary antibodies is shown in **Table 6** (**Supplementary Materia**l). The cells were incubated with primary antibodies diluted in PBS containing 0.2% Tween-20 on coverslips overnight at 4°C, followed by incubation with fluorescently labeled secondary antibodies for 2 h at RT. 0.2% PBS-Tween-20 (v/v) was used for all washing steps. Nuclei were visualized by adding 4,6-diamidino-2-phenylindole (DAPI) (Roche Diagnostics, Mannheim, Germany) (0.1 µg/mL) for 2 min at RT. Coverslips were mounted to the glass slides using Mowiol (Calbiochem, San Diego, CA, USA). The images were taken with an Axiocam digital camera attached to the Axio Observer Z1 microscope (Carl Zeiss Microscopy GmbH, Jena, Germany), using appropriate filters.

### 4.12 Chromatin immunoprecipitation

After reaching a confluence of 70%, the αTC1-6 cell’s chromatin was crosslinked with 1% formaldehyde (Zorka Pharma, Serbia) and the process was stopped with a glycine solution after 5 min at room temperature. Chromatin immunoprecipitation was completed using the Pierce™ Magnetic ChIP Kit (Thermo Fisher Scientific, USA) according to the manufacturer’s protocol. Cells were incubated for 10 min on ice in the buffer for cell extraction supplemented with a protease and phosphatase inhibitor cocktail. The cell nuclei sediment was incubated for 15 min at 37°C in digestion buffer with 0.01 U/µL micrococcal nuclease, after which they were subjected to sonication on ice (5 rounds of 20 s sonication and 20 s rest). Immunoprecipitation was performed with the following antibodies: normal rabbit IgG (supplied within the ChIP kit), anti-RNA polymerase II (RNA pol II) (supplied within the ChIP kit), 5 µg of anti-H3K4me3 (ab12209, Abcam, UK) and 5 µg of anti-H3K9me3 (ab8898, Abcam, UK) antibody overnight at 4°C. Samples were then incubated with Protein A/G Magnetic Beads for 2 h at 4°C with mixing. After elution for 30 min at 65°C min, the immunoprecipitated sample and 10% of total input were incubated with proteinase K for 90 min at 65°C. Purified DNA from immunoprecipitated samples was used for the evaluation of the abundance of the target sequence by quantitative PCR. The Maxima SYBR Green/ROX qPCR Master Mix (Thermo Fisher Scientific, USA) and QuantStudio 3 Real-Time PCR system (Applied Biosystems, Carlsbad, CA, USA) were used for PCR reaction. The set of primers for ChIP amplicon (**Table 7**, **Supplementary Material**) was designed to amplify part of the Arx promotor including the TSS (marked as +1) encompassing sequence from -189 to +25. Firstly, a standard curve was generated with qPCR data of 10-fold dilution series of the 10% total input samples. The enrichment was calculated by normalizing to IgG sample quantity and the fold enrichment was expressed relative to the NIT-1 samples immunoprecipitated by the same antibody.

### 4.13 Enzyme-linked immunosorbent assay

The amount of insulin released into the medium was measured using an enzyme-linked immunosorbent assay (ELISA) kit (EMD Millipore, St. Charles, Missouri, USA) according to the manufacturer’s instructions. For measuring the amount of released glucagon into the medium glucagon ELISA kit (Glucagon Quantikine ELISA Kit, R&D systems, Bio-Techne, USA) was used according to the manufacturer’s guidelines: the medium in which the cells were grown for 24 h was harvested and centrifuged at 500 x g for 10 min and 10 μL of the supernatant was used for measuring concentration of released hormones. The absorbance measurement was performed at the ELISA reader (Sunrise Basic, Tecan Austria GmbH, Grödig, Austria). For glucose-stimulated insulin secretion (GSIS) αTC1-6 and NIT-1 cells alone, or αTC1-6 mixed with NIT-1 cells in different ratio (0-10%) were used. Cells were incubated in Krebs-Ringer bicarbonate (KRB) buffer without glucose (116 mM NaCl, 1.8 mM CaCl_2_·2(H_2_O), 0.8 mM MgSO_4_·7(H_2_O), 5.4 mM KCl, 1 mM NaH_2_PO_4_·2(H_2_O), 26 mM NaHCO_3_ and 0.5% BSA, pH 7.4) for 1 h at 37°C. Cells were washed and incubated in KRB buffer with 30 mM glucose for 2 h at 37°C. After incubation, cell medium was collected and used for quantification of released insulin after glucose stimulation by ELISA assay. The used cell growth medium does not contain any traces of insulin and it was also used as additional blank during ELISA assay.

### 4.14 Statistical analysis

The GraphPad Prism 5 software for Windows (GraphPad Software, La Jolla, CA, USA, www.graphpad.com) was used for data analysis. Experiments were performed in three biological replicates (unless otherwise indicated) and presented as mean values ± SDs. A p-value less than 0.05 were considered statistically significant (∗p <0.05; ∗∗p <0.01; ∗∗∗p <0.001, ∗∗∗∗p <0.0001). The Kolmogorov-Smirnov test was used for determining normality of the sample. The statistical significance was established by using one sample t-test for normally distributed values. For the data with non-normal distribution, the Wilcoxon Signed Rank test was applied. An unpaired Student’s t-test was used to compare the mean values of the variables between the two groups.

## Data availability statement

The datasets presented in this study can be found in online repositories. The names of the repository/repositories and accession number(s) can be found below: http://www.ncbi.nlm.nih.gov/bioproject/922343. Accession number: PRJNA922343.

## Author contributions

Conceptualization, TJ, MV and JJ; methodology, MĐ, PS, CF and VP; validation, MĐ; CG and JR; formal analysis, MĐ, AT, CF, RJ and AU; investigation, MĐ, and MM; writing—original draft preparation, MĐ; writing—review and editing, MV, CG, TJ and JJ; visualization, MĐ, NG and SD; supervision, JJ; funding acquisition, MV and TJ. All authors have read and agreed to the published version of the manuscript.
