# Supplementary material for: EpiCRISPR targeted methylation of Arx gene initiates transient switch of mouse pancreatic alpha to insulin-producing cells
Source: Front Endocrinol (Lausanne). 2023 Mar 16;14:1134478. doi: 10.3389/fendo.2023.1134478 (PMC10063207; doi:10.3389/fendo.2023.1134478)
Supplement: Supplementary file 1 [file DataSheet_1.pdf]

Supplementary Material

Article

EpiCRISPR targeted methylation of Arx gene initiates transient switch of mouse pancreatic alpha to insulin-producing cells

Marija Đorđević[1], Peter Stepper[2], Clarissa Feuerstein-Akgoz[3], Clarissa Gerhauser[3], Verica Paunović[4], Anja Tolić[1], Jovana Rajić[1], Svetlana Dinić[1], Aleksandra Uskoković[1], Nevena Grdović[1], Mirjana Mihailović[1], Renata Z. Jurkowska[5], Tomasz P. Jurkowski[5*] Jelena Arambašić Jovanović[1*], Melita Vidaković[1¥]

Correspondence: jurkowskit@cardiff.ac.uk; TJ, jelena.arambasic@ibiss.bg.ac.rs; JAJ., melita@ibiss.bg.ac.rs; MV.

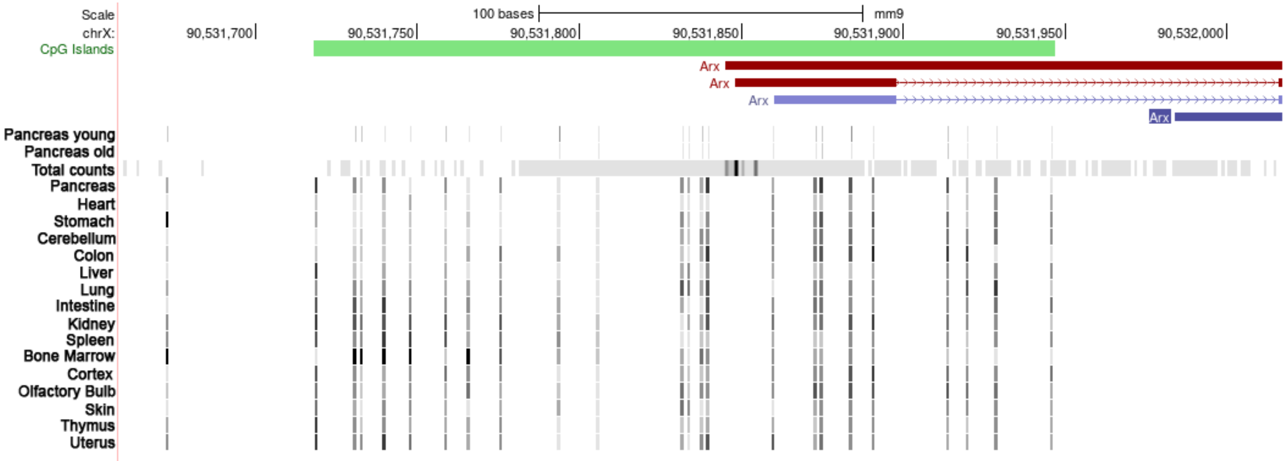

Supplementary Figure 1. UCSC genome browser views of murine Arx gene's DNA methylation sites in the pancreas and other tissues. If compared with other tissues, Arx gene in pancreatic tissue (young and old) exhibits low DNA methylation. CpG island was shown as a green box.

Supplementary Tables

Table 1. Targeted sequences for sgRNAs

		Targeting sequences of sgRNAs
**Arx sgRNA**	**1**	GCAAAGCGCAAAGCGCGAAC
	**2**	TAACAAGTGTAGTGAGCCGC
	**3**	AGGGTGGGAGCCCGCAACCG
	**4**	GATGCTGTCGATGCAGTAGG

Table 2. Primers used for RT-qPCR

Gene		Primer sequence (5'-3')
**REEP 5**	fw	TCATCGGACTGGTGGCTTTG
	rev	GTTGGGACTCTCGATGGCTT
**Cas 9**	fw	TCAGGCGGCAAGAGGATTTC
	rev	AGTCATCCACGCGAATCTGG
**Arx**	fw	CGGAGAAGCCCATGCAAAAT
	rev	TGCAGCTCAGCCTCGAAC
**Pax 4**	fw	GCTCGAATTGCCCAGCTAAAG
	rev	TTACTGTGGGGACTGGGAAGA
**Ins 2**	fw	GGAGCGTGGCTTCTTCTACA
	rev	TTCATTGCAGAGGGGTAGGC
**Slc2a2**	fw	TGCACCATCTTCATGTCGGT
	rev	ATTGCAGACCCAGTTGCTGA
**Maf A**	fw	TTCTGGAGAGCGAGAAGTGC
	rev	ACAGAAAGAAGTCGGGTGCG
**Pax6**	fw	TGTCTACCAGCCAATCCCAC
	rev	CATTCACTGACGGGCTGGT
**Pdx1**	fw	CCTTTCCCGAATGGAACCGA
	rev	GGGCCGGGAGATGTATTTGT
**Nkx2-2**	fw	TTCCGGACACCAACGATGAA
	rev	CCGTGCAGGGAGTATTGGA
**Nkx6-1**	fw	GAGAGCACGCTTGGCCTATT
	rev	TCGTCGTCATCCTCCTCATTC

Table 3. Primers used for HRM

Primers			Sequence (5'-3')	Primer length	Amplicon length
**R1**	M	Fw	AATTTTAAGGATAAGAAGGAGCGATA	26	198
		Rev	CGAACTACGTAAACCTACCG	20	
	U	Fw	TTTTAAGGATAAGAAGGAGTGATA	24	200
		Rev	AAACCAAACTACATAAACCTACCAA	25	
**R2**		Fw	GTATTAGGAAGAGGGTTGTTTTGAG	25	287
		Rev	ATATCTCCAACTTAACAAATCCCAA	25	

Table 4. Touchdown PCR program for amplifcation of bisulfte converted DNA, starting at 55 °C.

bisulfite PCR program		
**95**	**15 min**	
**94 °C**	**30 s**	**6 x**
**x °C**	**30 s**	**10 x**
**72 °C**	**40 s**	
**94 °C**	**30 s**	
**x-5 °C**	**30 s**	**35 x**
**72 °C**	**40 s**	
**94 °C**	**30 s**	
**x-10 °C**	**30 s**	
**72 °C**	**40 s**	
**72 °C**	**4 min**	
**4 °C**	**hold**	

Table 5. Primers for NGS library preparation

Primers	Sequence (5'-3')
**mArx_bis_1_f**	TTT TTT GTT TTT ATA TTT ATA GGT TTT TTT T
**mArx_bis_1_r**	TTA CTC ATA ACT AAT ACT TTT TCC TTA AAC
**mArx_bis_2_f**	GTT TAA GGA AAA AGT ATT AGT TAT GAG TAA
**mArx_bis_2_r**	TAT CTC CAA CTT AAC AAA TCC CA
**mArx_biscg2_3_f**	AGG TTG AGT TGT ATT TGT TAT TTA AGT
**mArx_biscg2_3_r**	CTA ACT AAT CTT AAA CRT ATC CCA AAC
**mArx_biscg2_4_f**	GTT TGG GAT AYG TTT AAG ATT AGT TAG
**mArx_biscg2_4_r**	AAT ACA ACA ACA ACT CTT CCT TAA A

Table 6. Antibodies used for Immunoblot analysis (IBA) and Immunocytochemistry (ICC)

	Antibody	Host species	Dilution IBA/ICC	Supplier
**Primary antibody**	anti-glucagon	Goat	1:500 / 1:50	C-18 / Santa Cruz Biotechnology
	anti-insulin	Rabbit	1:500 / 1:200	H-86 / Santa Cruz Biotechnology
	anti-Arx	Rabbit	1:1000 / 1:100	ab48856 / Abcam
	anti-beta Actin	Rabbit	1:2000 /1: -100	ab8227 / Abcam
**Secondary antibody**	anti-rabbit IgG-HRP	Cow	1:2000 / -	sc-2379 / Santa Cruz Biotechnology
	anti-goat IgG-HRP	Donkey	1:2000 / -	sc-2020 / Santa Cruz Biotechnology
	anti-goat IgG, F(ab')2-TRITC	Donkey	1:100	sc-3855 / Santa Cruz Biotechnology
	anti-rabbit IgG (H+L), Alexa Fluor 555	Donkey	1:4000	A-31572 / Invitrogen

Table 7. Primers for PCR reaction after ChIP

		Sequence (5’-3’)	Amplicon length
**ChIP primers**	fw	TTAGGGTTTGCCACCCCATCTA	208
	rev	CAATCAGCCTCCCCAAATGAAG	
